# Detection of canine obstructive nasal disease using infrared thermography: A pilot study

**DOI:** 10.1371/journal.pone.0291440

**Published:** 2023-09-12

**Authors:** Tekla Lee-Fowler, Stuart Clark-Price, Kara Lascola

**Affiliations:** Department of Clinical Sciences, Auburn University, Auburn, AL, United States of America; University of Wisconsin-La Crosse, UNITED STATES

## Abstract

Infrared thermography detects variations in heat signature and is utilized in other species to non-invasively identify respiratory disease. This study aimed to determine if infrared thermography could be used to detect nasal disease in dogs. Eight dogs presenting for nasal disease (ND group) and ten healthy control dogs (C group) were enrolled. Dorsal and rostral images of the nose were acquired using a Fluke TiX580 60Hz thermal imaging camera. Images were analyzed using the accompanying software. Regions of interest were defined over the right and left nasal passages to determine the maximum (max), average (avg), and minimum (min) temperatures. Temperatures were compared between ND and C groups, and correlation to disease state (ND or C) was evaluated. Temperature differences and imaging patterns were subjectively compared with diagnosis based on computed tomography (CT) and histopathology. The ND group consisted of 5 spayed females and 3 neutered males. Clinical sings included unilateral epistaxis (n = 4); bilateral serous discharge and sneezing (n = 1); bilateral mucopurulent discharge, epistaxis, and sneezing (n = 1); unilateral mucoid discharge, epistaxis and sneezing (n = 1); and sneezing and unilateral epistaxis (n = 1). Temperatures were significantly different between ND and C groups on dorsal (max *p* = <0.001, avg *p* = 0.001, min *p* = <0.001) and rostral (max *p* = <0.001, avg *p* = <0.001, min *p* = 0.005) images. Temperature positively correlated to disease status (ND vs C group) in both dorsal and rostral images. Subjective analysis of images allowed correct identification of abnormal or normal 27/36 times. Obstructive nasal disease results in a local temperature increase in the affected nasal passage that can be non-invasively detected by infrared thermography.

## Introduction

Infrared thermography (IRT) technology has been utilized in the medical field as a detection tool for diseases with features such as regional vasodilation, hyperperfusion, increased vascularization, increased metabolism, and hyperthermia [1]. IRT has been used in veterinary medicine to investigate a wide range of conditions including pain in laboratory animals, heat loss in newborn lambs, fever in swine, estrus detection in swine, physiologic responses to preslaughter handling in pigs, footpad pathologies in chickens, fever detection in birds with avian influenza, stress in birds, limb and hoof health and lameness in cattle and horses, pregnancy detection in horses, mastitis in dairy cows, and hyperthyroidism in cats [2–22]. In dogs, IRT has been used to investigate various conditions including but not limited to orthopedic disease, neurologic disease, neoplastic disease, and anesthetic effects on body temperature, and physiotherapy exercise [23–30].

Infrared thermal imaging cameras non-invasively measure heat signature, or energy in the infrared wavelength, and convert it into a visible light image. These cameras are now widely available and can be acquired at relatively minimal cost. Additionally, they have the advantage of being portable, with some models integrating with mobile devices, and they are easy to use. The process of acquiring an IRT image is similar to acquiring an image with a digital camera, and images can be viewed in real-time and downloaded to computer software that accompanies the camera.

Obstructive nasal disease, such as occurs with nasal congestion, increases the temperature of the nasal passage [31]. This has traditionally been evaluated through direct instrumentation of the nasal passage with a thermocouple and rhinomanometry. However, studies utilizing infrared thermography for evaluation of the nasal passage in humans with chronic sinusitis and nasal septal perforations demonstrated the utility of IRT for diagnosis of these conditions [32, 33].

The goal of this study was to determine if images acquired with an infrared thermal imaging camera could be used to distinguish between dogs with obstructive nasal disease and healthy dogs. The study evaluated both subjective data, such as the image that would be available real-time, and objective data, which requires temperature analysis with accompanying software. We hypothesized that IRT would allow for the determination of nasal airflow obstruction and differentiation between diseased and healthy dogs.

## Materials and methods

### Animals

Canine patients presenting to the Auburn University Bailey Small Animal Teaching Hospital for evaluation of signs of nasal disease, including nasal discharge, epistaxis, and sneezing were recruited for this study. Patients were included in this nasal disease group (ND) if planned diagnostics were to include nasal computed tomography exam (CT) and biopsy to determine the underlying etiology of the clinical signs. A group of healthy dogs with no history or physical exam findings of respiratory disease were recruited as a control population (C). Client consent was acquired for all participants, and this study was approved by the Auburn University Institutional Animal Care and Use committee (#2019–3486).

### Image acquisition & analysis

IRT images were acquired using a Fluke TiX580 60Hz portable thermal imaging camera (Fluke Thermography, Plymouth, MN). This camera has a 640 x 480 pixel detector and spectral range of 7.5μm—14μm. An emissivity value of 0.95 was used. Room temperature was measured prior to patient image acquisition by acquiring an image of a non-reflective surface within the room (i.e. the wall). A period of acclimatization to the room occurred prior to image acquisition. Patient images were acquired with the camera placed at a 1.5–2 feet distance from the dog with the dog standing in a neutral position. In order to ensure no heat transfer occurred, handlers did not touch the animal’s face prior to imaging. For patients in the ND group, all images were acquired at the beginning of the visit prior to any diagnostics being performed. Dorsal and rostral images of the face were obtained with light restraint of the patient.

**Fig 1:**
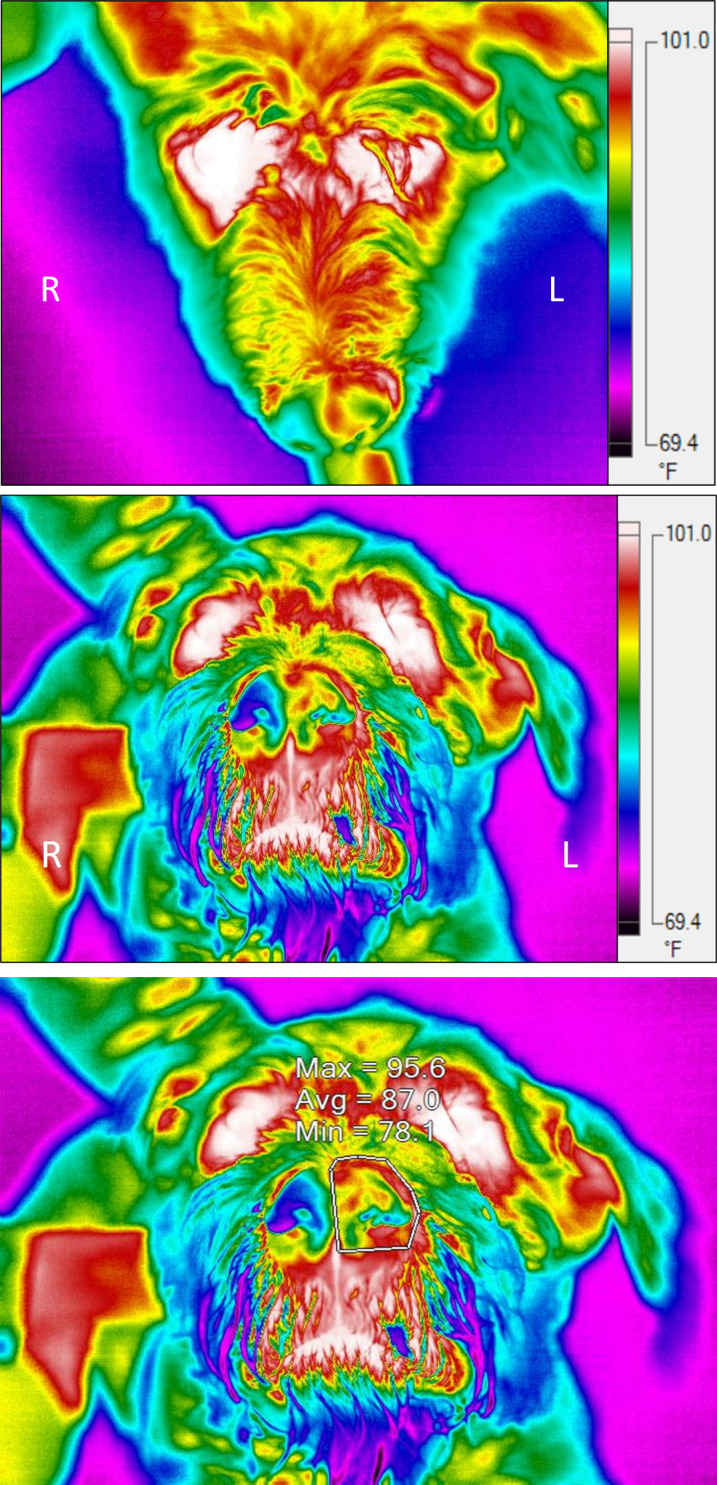
Thermal image examples. Dorsal (a) and rostral (b) IRT images of a dog diagnosed with a left sided nasal mass. The higher temperature of the left nasal passage can be subjectively appreciated. In image (c), a region of interest (ROI) has been identified on the rostral image with maximum (max), average (avg), and minimum (min) temperatures within this area displayed.

Images were analyzed in the high contrast color scale using the accompanying software, SmartView 4.3 (Fluke Thermography). Regions of interest (ROI) were manually drawn over the right and left side of the nose on both the dorsal and rostral images using freeform drawing tools in the software (Fig 1). Maximum (max), average (avg), and minimum (min) temperatures within the ROI were recorded. The room temperature was recorded from the images of the wall within the room.

Diagnoses were recorded for each ND dog based on CT and biopsy results. A single investigator that was not involved in patient diagnostics or acquisition of the IRT images (KL) was provided with coded IRT images of both ND and C dogs for review. The images had not been analyzed, and therefore, contained no temperature information. The investigator was blinded to the dog’s group and diagnosis and was asked to assign the dog to either a normal or abnormal (nasal disease) category based solely on the appearance of the images. Additionally, if the investigator assigned the dog to the abnormal category, they were asked to indicate if the abnormality was located on the right or left side of the patient image. The categorization was compared to the group and diagnosis and assigned as either correct or incorrect by a second investigator (TLF).

### Statistical analysis

Statistical analysis was performed using SigmaPlot (Systat Software). Normality was assessed via a Shapiro-Wilk test. Temperatures (max, avg, and min) acquired from the right and left sides of the nose on both dorsal and rostral images were compared using a One-Way Analysis of Variance (ANOVA) when data were normally distributed and a Kruskal-Wallis One-Way ANOVA on Ranks with a Tukey Test for multiple comparison when data was not normally distributed. Based on this initial analysis, right and left sided data were combined, and further analysis was conducted on dorsal images and rostral images separately to compare temperatures between ND and C groups. This comparison was completed using a Mann-Whitney Rank Sum test. Room temperatures were compared using a t-test. Spearman correlation was used to evaluate the correlation between disease state (ND or C group) and temperatures (max, avg, min temperature) for dorsal and rostral images. Categorical data (ND group vs C group or abnormal vs normal) was converted to binary data (i.e. ND = 1, C = 0 and correct = 1, incorrect = 0) for analysis. Normally distributed data is presented as mean±SD, and non-normally distributed data is presented as median (range). A *p*<0.05 was used for significance.

## Results

### Animals

Ten dogs with clinical signs of nasal disease were initially evaluated for inclusion in the study. Two dogs were excluded due to advanced imaging (nasal CT) and biopsy not being pursued. Of the excluded dogs, one failed to return for imaging after initial enrollment, and the second dog’s owner elected to discontinue diagnostic evaluation after neoplastic cells were found on complete blood count. Enrolled dogs (n = 8) in the study (ND group) ranged in age from 2–13 years (9.6±3.2 years), and include 3 neutered, males and 5 spayed, females. Breeds included 3 mixed breed dogs, 2 goldendoodles, and 1 each of the following: French bulldog, miniature schnauzer, and golden retriever. Presenting complaints included unilateral epistaxis (4 dogs), combined bilateral serous discharge and sneezing (1 dog), combined bilateral mucopurulent discharge, epistaxis, and sneezing (1 dog), combined unilateral mucoid discharge, epistaxis and sneezing (1 dog), and combined sneezing and unilateral epistaxis (1 dog). Clinical signs ranged in duration from 5 to 180 days (88.6±73 days) prior to presentation. Six dogs were diagnosed with nasal neoplasia, 1 dog with lymphoplasmacytic rhinitis, and 1 dog with focal inflammation/granuloma. Ten healthy control dogs aged 2–13 years (6.9±3.8 years) with no history or clinical signs of nasal disease were also enrolled (C group); 6 neutered, males and 4 spayed, females were included. Represented breeds included 3 mixed breed dogs, 3 Labrador retrievers, 2 Dobermann pinschers, 1 Jack Russell terrier, and 1 hound.

### Image acquisition & analysis

Room temperatures were not significantly different between groups (*p* = 1.0). Image acquisition was well tolerated in all dogs with minimal restraint. A total of 36 images (18 rostral, 18 dorsal) were acquired. No significant difference was found in temperature between the right and left sides of the nose in either dorsal or rostral images for max, avg, and min temperatures. However, significant differences were noted between dorsal and rostral images (*p* = <0.001). Therefore, further analysis combined right and left sided data to evaluate for differences between ND and C groups but evaluated dorsal and rostral images separately. A statistically significant difference was found between ND and C groups for each temperature in dorsal (max *p* = <0.001, avg *p* = 0.001, min *p* = <0.001) and rostral (max *p* = <0.001, avg *p* = <0.001, min *p* = 0.005) images (Table 1). Temperature positively correlated to disease status (ND vs C group) in both dorsal and rostral images with the minimum temperature on the dorsal images most positively correlating to disease status (Table 2).

**Table 1:** Mean ± SD of maximum, average, and minimum temperatures obtained from dorsal and rostral images in dogs with nasal disease (ND group) and controls (C group).

	Nasal Disease			Controls			
	Mean		SD	Mean		SD	P value
	Dorsal views			Dorsal views			
	°F	°C		°F	°C		
Maximum temperature	96.34	35.74	3.15	92.36	33.53	2.25	<0.001
Average temperature	86.99	30.55	2.86	83.15	28.42	2.85	0.001
Minimum temperature	77.04	25.02	5.99	68.88	20.49	2.5	<0.001
	Rostral views			Rostral views			
Maximum temperature	94.21	34.56	4.99	84.74	29.3	5.82	<0.001
Average temperature	83.54	28.63	4.96	74.63	23.68	4.55	<0.001
Minimum temperature	74.89	23.83	3.95	70.70	21.5	7.11	0.005

**Table 2:** Temperature positively correlated to disease status (nasal disease vs control) for both dorsal and rostral views. Correlation coefficients and p values are included for maximum, average, and minimum temperatures for both dorsal and rostral views.

	Dorsal views	P value	Rostral views	P value
Maximum temperature	0.616	0.0000654	0.616	0.000066
Average temperature	0.539	0.000762	0.678	0.00000239
Minimum temperature	0.813	0.0000002	0.495	0.00227

Images scored by the blinded investigator were correctly categorized as abnormal or normal 27/36 times with 14/18 dogs categorized correctly using the dorsal images and 13/18 dogs based on the rostral images. Of images that were incorrectly categorized, one dog with a nasal mass resulting in mild airflow limitation based on physical examination was misidentified as normal on both dorsal and rostral views. Another dog was categorized as normal when the rostral view was evaluated; however, this dog was identified correctly on the dorsal view. For images correctly assessed as abnormal, the abnormal side was successfully localized in all but one dog. In this dog, the side was only misinterpreted on the rostral view and was identified correctly on the dorsal view. This dog had rhinitis with mild, unilateral airway obstruction. Three images from dogs in group N were incorrectly categorized as abnormal based on dorsal images, and two were incorrectly categorized as abnormal on the rostral images; however, these errors only overlapped (both images incorrectly identified as abnormal) in a single dog.

## Discussion

The results from this study suggest that infrared thermography may be useful in detection of obstructive nasal disease in dogs. In this study, obstructive nasal disease resulted in an increase in the temperature of the affected side detected via IRT. In many cases, subjective assessment of the images, which can occur in real time, can identify obstructive nasal disease and determine which side is more clinically affected. Dorsal and rostral views appear to complement one another, and it is recommended to acquire both views in order to provide the best assessment. While evaluating maximum, average, and minimum temperatures can all be useful in suggesting when obstructive nasal disease is present, the minimum temperatures acquired on the dorsal images were most positively correlated with disease state.

One of the key functions of the nasal passage is warming of inspired air. This is accomplished through evaporative cooling from the surface of the nasal mucosa. Factors influencing nasal mucosal temperature include core body temperature, blood flow through the nasal mucosal blood vessels, rate of airflow through the nasal passages, and inspired air temperature [34]. Therefore, it is reasonable to consider that conditions resulting in decreased airflow as well as increased blood flow through the nasal passages may result in increased temperature of the nasal passages. Additionally, the angiogenesis associated with neoplastic masses and/or inflammation associated with such lesions may contribute further to temperature increase [1]. Visual identification of disease was possible in many cases simply by evaluating the color differences on acquired IRT images suggesting a difference in temperature. Dogs with nasal disease that did not appear to result in airflow limitation in this study were less easily identified on subjective analysis. It was not possible in this study to determine if this is more easily accomplished with unilateral or bilateral disease; however, the comparison of symmetry between sides would make it likely that unilateral disease is most easily detected.

Both dorsal and rostral views of the nose were evaluated in this study. When both views were subjectively evaluated, only two dogs (ND = 1, C = 1) were incorrectly categorized. One of these dogs had a mild decrease in airflow noted on physical exam (present but decreased air movement), and a nasal mass was diagnosed on CT. Objective analysis of this dog’s max, avg, and min nasal temperatures would also not have improved categorization. The mild nature of the airflow limitation could have contributed to the difficulty in subjectively assessing this dog. The other dog was in the control group and was categorized as abnormal based on subjective evaluation. The dog had no history or clinical signs of nasal disease; however, control dogs did not undergo CT imaging. Therefore, it was not possible to assess if there was in fact occult nasal disease present. Based on these findings, routine acquisition and evaluation of both views is recommended to maximize accuracy, particularly when subjectively evaluating the images. The infrared camera software provides analysis of the max, avg, and min temperatures within a region of interest. All temperatures were significantly different when comparing dogs with nasal disease and control dogs; therefore, based on the data from this study, any one of these could be used. However, the low temperature acquired on the dorsal images correlated most strongly with the disease status, and it would be recommended to evaluate the low temperature first on this view.

IRT is a non-invasive, portable tool that could be used in the clinical setting. IRT cameras are relatively low cost and becoming more affordable with some integrating with mobile phones. IRT has been utilized in other areas of veterinary medicine [23–29], and some practices may already be utilizing this equipment. This study was an initial, pilot investigation to determine if infrared thermal imaging could be utilized to detect disease in the nasal cavity, and obstructive nasal disease was of the most interest due to the potential difference in temperatures based on airflow limitation. Further study is needed to determine if this tool can be used to differentiate between nasal diseases, particularly infectious, inflammatory and neoplastic conditions. A low number of dogs in this study with mild airflow limitation could have contributed to the difficulty of differentiating this circumstance from normal dogs. As this study was designed, it was not possible to investigate dogs with a mild airflow limitation separately. It is not recommended that IRT be used as a stand-alone diagnostic tool. Based on this study, IRT can provide information that is complimentary to the current initial evaluation (i.e. history, physical examination) to help determine which animals are more likely to have an obstructive nasal disease.

## Conclusions

IRT can be a complimentary tool to aid in identification of obstructive nasal disease in the dog. Both subjective and objective evaluation of acquired images can be utilized. Both dorsal and rostral images should be evaluated, and objective evaluation of both high and low temperatures are recommended.

## Supporting information

S1 Fig Thermal imaging data. (XLSX)
